# Bedside Ultrasound for Hemodynamic Monitoring in Cardiac Intensive Care Unit

**DOI:** 10.3390/jcm11247538

**Published:** 2022-12-19

**Authors:** Maria Concetta Pastore, Federica Ilardi, Andrea Stefanini, Giulia Elena Mandoli, Stefano Palermi, Francesco Bandera, Giovanni Benfari, Roberta Esposito, Matteo Lisi, Annalisa Pasquini, Ciro Santoro, Serafina Valente, Antonello D’Andrea, Matteo Cameli

**Affiliations:** 1 Department of Medical Biotechnologies, Division of Cardiology, University of Siena, 53100 Siena, Italy; 2 Department of Advanced Biomedical Sciences, University of Naples Federico II, 80138 Naples, Italy; 3 Mediterranea Cardiocentro, 80122 Naples, Italy; 4 Public Health Department, University of Naples Federico II, 80131 Naples, Italy; 5 Cardiology University Department, Heart Failure Unit, IRCCS Policlinico San Donato, San Donato Milanese, 20097 Milan, Italy; 6 Department of Biomedical Sciences for Health, University of Milano, 20122 Milan, Italy; 7 Section of Cardiology, Department of Medicine, University of Verona, 37129 Verona, Italy; 8 Department of Clinical Medicine and Surgery, Federico II University Hospital, 80131 Naples, Italy; 9 Department of Cardiovascular Disease—AUSL Romagna, Division of Cardiology, Ospedale S. Maria delle Croci, Viale Randi 5, 48121 Ravenna, Italy; 10 Department of Cardiovascular and Thoracic Sciences, Fondazione Policlinico Universitario A. Gemelli IRCCS, Università Cattolica del Sacro Cuore, 20123 Rome, Italy; 11 Department of Cardiology, Umberto I Hospital, 84014 Nocera Inferiore, Italy

**Keywords:** intensive care unit, echocardiography, lung ultrasound, vexus, pulmonary hypertension

## Abstract

Thanks to the advances in medical therapy and assist devices, the management of patients hospitalized in cardiac intensive care unit (CICU) is becoming increasingly challenging. In fact, Patients in the cardiac intensive care unit are frequently characterized by dynamic and variable diseases, which may evolve into several clinical phenotypes based on underlying etiology and its complexity. Therefore, the use of noninvasive tools in order to provide a personalized approach to these patients, according to their phenotype, may help to optimize the therapeutic strategies towards the underlying etiology. Echocardiography is the most reliable and feasible bedside method to assess cardiac function repeatedly, assisting clinicians not only in characterizing hemodynamic disorders, but also in helping to guide interventions and monitor response to therapies. Beyond basic echocardiographic parameters, its application has been expanded with the introduction of new tools such as lung ultrasound (LUS), the Venous Excess UltraSound (VexUS) grading system, and the assessment of pulmonary hypertension, which is fundamental to guide oxygen therapy. The aim of this review is to provide an overview on the current knowledge about the pathophysiology and echocardiographic evaluation of perfusion and congestion in patients in CICU, and to provide practical indications for the use of echocardiography across clinical phenotypes and new applications in CICU.

## 1. Background

Patients in the cardiac intensive care unit (CICU) are frequently characterized by conditions that rapidly change and evolve into several clinical phenotypes connected with the underlying etiology and its complexity; phenotyping patients helps to early guide the appropriate management strategies towards the underlying etiology. In these patients with signs of hemodynamic instability, echocardiography is the most reliable bedside method to assess cardiac function repeatedly, assisting clinicians not only in characterizing hemodynamic disorders, but also in helping to guide interventions and monitor response to therapies (such as intravenous fluids, inotropes, and ultrafiltration). Standard echocardiographic evaluation should rapidly provide sufficient information to confirm/exclude tamponade, mechanical complications of acute myocardial infarction (AMI), left ventricular (LV) outflow tract obstruction (LVOTO), severe valvular lesions and LV and right ventricular (RV) dysfunction [1,2,3].

The goal of this review is to summarize the current knowledge concerning the definition, pathophysiology and echocardiographic evaluation of perfusion and congestion in patients in the CICU, also based on recent tools such as lung ultrasound (LUS) and the Venous Excess UltraSound (VexUS) grading system. It will also detail another application of echocardiography in the CICU based on the evaluation of the presence of pulmonary hypertension, both as an additional element for the assessment of the patient’s hemodynamics, and to guide the setting and modifications of ventilatory oxygen therapy.

## 2. Evaluation of Perfusion

### 2.1. Pathophysiology in Intensive Care Unit

Only a minority of patients in the CICU with acute heart failure (HF) present with signs and symptoms of low perfusion correlated to low cardiac output (CO). Cardiac insult causing the severe impairment of cardiac performance may be acute, as a result of an acute coronary syndrome (ACS) (80% of cases of cardiogenic shock) [4,5] or may be progressive, as in patients with a progression of chronic decompensated HF [6]. Ineffective stroke volume goes to determine a compensatory peripheral vasoconstriction that initially improves coronary and peripheral perfusion, but it contributes to increased cardiac afterload that overloads and aggravates heart conditions.

A state of perfusion that is inadequate to meet the metabolic demands of tissues results in hypoxia and inadequate aerobic metabolism and, ultimately, systemic inflammation, cell injury, cell death, tissue injury and organ failure [4]. Cold sweated extremities, oliguria, mental confusion, dizziness and narrow pulse pressure are the main clinical signs of hypoperfusion; moreover, reduced kidney function, metabolic acidosis and elevated serum lactate are present and reflect tissue hypoxia and alterations of cellular metabolism leading to organ dysfunction [6].

Acute HF with a clinical profile of low CO is usually associated with low systolic blood pressure (BP) (<90 mmHg) and mean arterial pressure (<65 mmHg) [4]. Of note, hypoperfusion is not always accompanied by hypotension, as BP may be preserved by compensatory vasoconstriction (with/without pressor agents), at the cost of impaired tissue perfusion and oxygenation [6,7]. Besides, the true perfusion pressure of an organ is in fact not the mean arterial pressure (MAP) minus central venous pressure (CVP), but rather the precapillary arteriolar pressure minus postcapillary venular pressure; the precapillary arteriolar pressure is substantially lower than the large arterial pressures. Hence, arterial BP is indeed critically important, but the venous side also has an important impact on organ function [8].

### 2.2. Echocardiographic Variables

**Figure 1:**
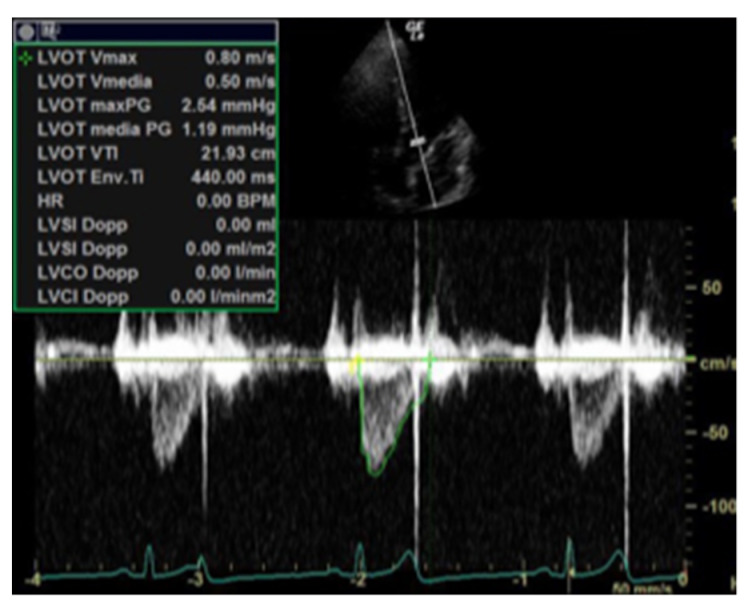
LVOT Velocity Time Integral (VTI), which is the distance the blood travels across the LVOT, calculated after tracing the LVOT pulsed−wave doppler.

First of all, perfusion can be evaluated through LV Ejection Fraction (EF), but it is well known that this parameter is greatly influenced by LV geometry, heart rate and loading conditions (preload and afterload). As a result of that, its usefulness is limited in critically ill patients [2]. Hence, in severe shock states, the estimation of CO, measured as a calculation of stroke volume (SV) times the heart rate, is a more reliable quantitative parameter as it truly reflects circulatory status and organ perfusion [9]. In the standard method, SV is determined by the LVOT area (obtained by measuring the LVOT diameter in parasternal long-axis view in mid-systole), multiplied by the LVOT Velocity Time Integral (VTI)—which is the distance the blood travels across the LVOT—calculated after tracing the LVOT pulsed-wave doppler (PWD) spectral display (Figure 1). Moreover, cardiac index (CI) normalizes flow (CO) with the body surface area (BSA). Normal LVOT VTI is higher than 18 cm and it could be used as a surrogate for SV, which is also an important predictor of fluid responsiveness. In particular, there are static measures of fluid responsiveness (inferior vena cava (IVC) diameter, CI and left ventricular end-diastolic area (LVEDA)) and dynamic measures (IVC distensibility in spontaneous breath and LVOT VTI respiratory variability) [2,7,10].

Furthermore, LV global systolic function could be examined through peak global longitudinal strain (GLS) that describes the relative length change of the LV myocardium between end-diastole and end-systole. This parameter is less used in the context of critical ill patients, but it is a valuable and sensitive tool for follow-up examinations. LV GLS should be made in the three standard apical views and averaged, after optimizing image quality, maximizing the frame rate and minimizing foreshortening (a peak GLS in the range of −20% can be expected in a healthy person, even though reference values vary according to age and sex) [11,12]. In patients hospitalized for acute HF, acute myocardial infarction, myocarditis, the values are lower (ranging from −4% to −17%) [13,14,15]. In fact, a recent study suggested the use of LV GLS −17% as diagnostic cut-off values in patients with sepsis or septic shock [16]. However, there currently is a lack of international standardization for strain values in different pathologic conditions.

Moreover, being more sensitive to acute changes in cardiac loading conditions than conventional echocardiographic parameters, LV GLS may have another important application in ICU. In particular, the evaluation of LV strain in acute HF has the potential to define treatment responsiveness among different clinical phenotypes [17].

Likewise, the echocardiographic assessment of RV in critically ill patients is important. First, it should include the assessment of the right ventricle outflow tract (RVOT) VTI that gives information about the RV function and pulmonary vascular resistance (PVR), even if the echocardiographic measurement of PVR (through the ratio between tricuspid regurgitant jet velocity (TRV) and RVOT VTI [18]) is not fully validated to initiate or monitor treatment of pulmonary hypertension (PH), and should not replace invasive measurement by right heart catheterization (RHC) [19]. Secondly, the assessment of RV should include RV size and systolic function as evaluated by: RV Fractional Area Change (FAC), which provides an estimate of global RV systolic function (if <35% indicates RV systolic dysfunction); and Tricuspid Annular Plane Systolic Excursion (TAPSE), that represents a measure of RV longitudinal function and the tricuspid lateral plane systolic velocity by TDI (S’), which has been shown to correlate well with other measures of global RV systolic function (if <9.5 cm/s, indicates RV systolic dysfunction). Moreover, RV longitudinal strain is a useful parameter for estimating RV global and regional systolic function and is calculated as the percentage of systolic shortening of the RV free wall from base; it is less influenced by overall heart motion, but depends on RV loading conditions as well as RV size and shape. Peak global longitudinal RV strain excluding the interventricular septum has prognostic value in heart failure, acute myocardial infarction and pulmonary hypertension [11].

### 2.3. Echocardiography: Dynamic Indices

**Figure 2:**
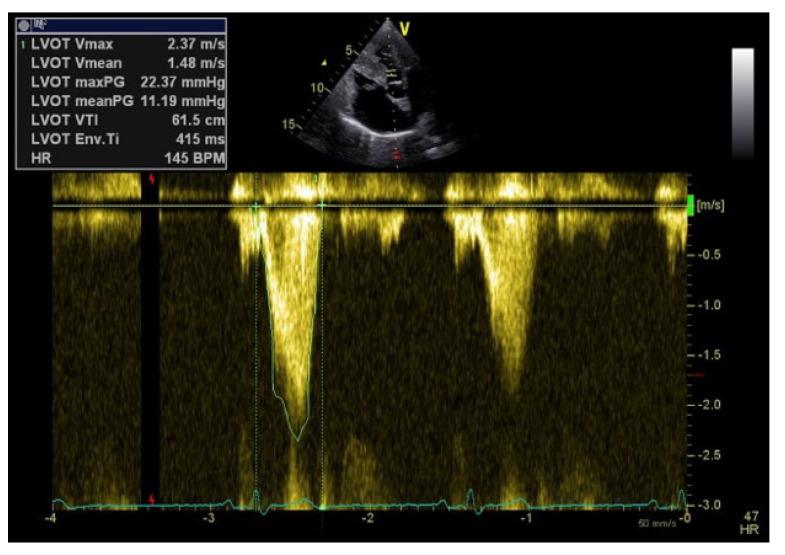
Typical morphological appearance of the Doppler signal “dagger−shaped” and late peaking curve in left ventricular outflow tract obstruction.

The assessment of perfusion should also include the possible presence of dynamic LVOTO in which certain anatomical variations facilitate this phenomenon: the small diameter of the LVOT due to basal septal hypertrophy; elongated anterior mitral leaflet; and the presence of systolic anterior motion of the anterior mitral leaflet with leaflet-septum contact in mid-systole. A continuous Doppler is used to measure the degree of obstruction and pulsed Doppler to locate the exact point where the obstruction is occurring. The typical morphological appearance of the Doppler signal is a “dagger-shaped” and late peaking curve (Figure 2). This is because at the beginning of systole, ejection begins normally, but as systole progresses the outflow tract narrows due to increased pressure and the pressure increases further due to the narrowing of the outflow tract. Thus, the continuous Doppler shows a progressive flow acceleration pattern. Significant LVOTO is defined as a peak gradient ≥30 mmHg (rest or provoked trough Valsalva manoeuvre) [20]. The echocardiographic diagnosis of dynamic LVOTO or LV mid-cavitary obstruction often leads to drastic changes in the clinical management as it entails stopping or reducing inotropes, increasing afterload by vasopressors, optimizing preload by administering intravenous fluids and also reducing heart rate with drugs or pacing optimization [2].

## 3. Congestion: Diagnosis and Monitoring

### 3.1. Pathophysiology in Intensive Care Unit

The vast majority of acute heart failure (AHF) episodes are characterized by increasing symptoms and signs of congestion. Congestion has a pivotal role in critically ill patients and it results in increased cardiac filling pressures that determine signs and symptoms of extracellular fluid accumulation [21]. Filling pressure depends on venous compliance/capacitance, plasma volume and cardiac function. Indeed, the transition from chronic to AHF is often attributed to increased sodium avidity and extracellular volume overload. However, it has also been suggested that the redistribution of volume may be a frequent cause of increased cardiac filling pressures; splanchnic arterioles and veins are very sensitive to changes in sympathetic activity. Increased sympathetic output leads to splanchnic arterial and venous constriction and blood redistribution from the splanchnic capacitance vasculature to circulatory volume, which increases venous return and raises cardiac filling pressures [4,22,23].

The raise in preload by congestion leads to increased ventricular wall stress, valvular regurgitation, myocardial stretch, remodeling, ventricular myocyte necrosis and a progressive decline in cardiac function [24]. Moreover, natriuretic peptides are released due to these conditions.

It is well known the importance of detecting and monitoring congestion before it leads to decompensation, is a clear predictor of poor outcome. However, the assessment of congestion can be difficult, especially when the extrapulmonary signs of congestion are mild, such as in the setting of acute pulmonary congestion due to hypertension. Increased intracardiac filling pressures often silently precede the appearance of congestive symptoms by days or weeks.

### 3.2. Echocardiography

Echocardiography provides detailed information about cardiac structure and function in acute cardiovascular disease, and remains the gold standard for evaluating blood volume and LV filling pressures [24].

**Figure 3:**
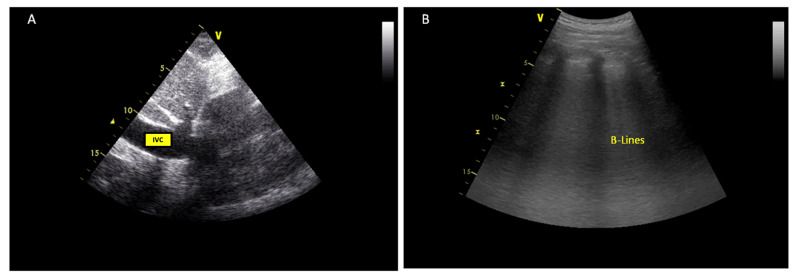
Echocardiographic markers of congestion. (**A**) increased IVC inferior vena cava (IVC) diameter; (**B**) lung ultrasound showing vertical B-lines as a measure of interstitial oedema.

Echocardiographic parameters can be used to estimate right- and left-sided filling pressures, although with less reliability in AHF. The estimation of right atrial (RA) pressures can be performed by assessing the diameter of the IVC and the percentage decrease in the diameter during inspiration. The diameter of the IVC decreases in response to inspiration when the negative intrathoracic pressure leads to an increase in RV filling from the systemic veins. IVC is commonly dilated and may not collapse in patients on ventilators, so it should not be routinely used in such cases to estimate RA pressure [25] (Figure 3).

Left-sided filling pressures assessment is performed by assessing the trans-mitral flow by PWD and then estimating mitral inflow E (early diastolic relaxation) and A (late atrial contraction) waves. TDI is then used to assess the mitral annulus diastolic velocities (e’ and a’). An increase in early diastolic mitral inflow velocities (E wave) occurs when filling pressure raise. This is indicative of increased filling pressures in the presence of a low e’ (lateral e’ < 8 cm/s was associated with increase mortality in the critically ill patients [26] and it provided a 12% increase in ICU-mortality risk per unit decrease in cm/s in another study [27]), especially if E-wave deceleration time is short and A-wave velocities are low [2]. E/e’ ratio (computed from the average of the septal and lateral e’) is considered an important load-independent marker of LV filling pressure and has been shown to correlate with pulmonary capillary wedge pressure (PCWP) in a wide range of cardiac patients [28]. Nevertheless, in decompensated patients with advanced systolic heart failure, E/E’ ratio should be used with caution because it may not be reliable in predicting intracardiac filling pressures, particularly in patients with larger LV volumes, more impaired cardiac output, and the presence of cardiac resynchronization therapy (CRT) [29]. Besides, there are other limitations of this index to consider: the majority of critically ill patients have an E/e’ in a “grey zone” between 8 and 14; mitral stenosis and severe mitral regurgitation invalidate the measurement of mitral inflow velocities; and positive pressure ventilation could influence LV filling pressure in several ways, making these indices unreliable [2,30].

**Figure 4:**
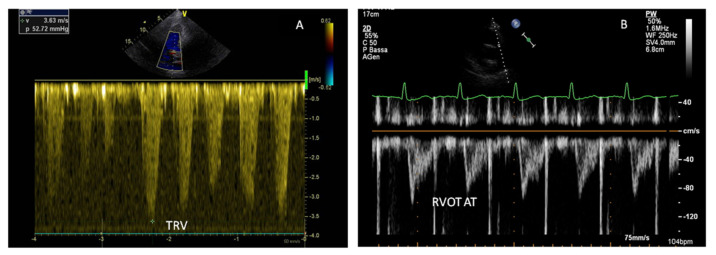
Echocardiographic markers of pulmonary hypertension (PH). (**A**) Increased peak tricuspid regurgitation velocity (TRV); (**B**) reduced right ventricular outflow tract (RVOT) acceleration time.

The analysis of pulmonary venous PWD waveforms, left atrial (LA) size and tricuspid regurgitation (TR) jet are also required for a comprehensive LV diastolic function assessment. In particular, echocardiography is the most used non-invasive tool for estimating pulmonary arterial pressure (PAP). Systolic PAP (sPAP) can be measured by adding the RAP to the TR peak systolic gradient, that derived from the TR peak systolic velocity. Moreover, the measurement of the pulmonary artery acceleration time (PAAT) could indicate an elevation in the PVR, which is demonstrated by shortening of the PAAT (normal PAAT interval values in adults range from 136 to 153 ms) with or without mid-systolic notching; PAAT, in fact, represents pulmonary flow acceleration, which increases as the vascular resistance is augmented, based on the Newton law of motion. Different studies have demonstrated a reasonable accuracy of PAAT in correctly estimating sPAP and mean PAP (mPAP). However, PAAT measurement to derive sPAP is not reliable in critically ill patients, particularly in the coexistence of RV systolic impairment [31,32] (Figure 4).

Finally, the left atrium influences LV filling and performance through its functions of contractile pump and reservoir. LA enlargement (preferentially measured by LA volume) is a marker of both the severity and chronicity of diastolic dysfunction and the magnitude of LA pressure elevation [11]. The evaluation of peak atrial longitudinal strain (PALS) by Speckle-tracking echocardiography is also useful as an index of LV filling pressures and diastolic function: this parameter was also tested in AHF patients and it was demonstrated that in this context, associated with N-terminal pro B-type natriuretic peptide (NT-proBNP), it may be used as an additional index of congestion to optimize therapeutic management and could enhance the prognostic stratification of HF [33] (Table 1).

A new landscape in cardiac imaging is represented by the application of artificial intelligence (AI) algorithms (particularly machine learning), that could enhance efficiency, precision and timeliness, which are of great relevance in the CICU. AI optimize the quality of images, endocardial tracing, Doppler representations and supports three-dimensional echocardiography, allowing the cardiologist to save time and help standardize reproducibility.

Moreover, AI aids to better define static, dynamics, planimetric and volumetric measurements of the valves, flows and regurgitations. Even though it is considered an expanding topic needing further research, AI is near to being fully integrated into clinical practice and its use could be of great utility in the CICU, where time-saving and precise evaluation are fundamental [34,35,36,37].

The use of bedside ultrasound in children may be quite different and should consider different aspects. First of all, data supporting the use of bedside ultrasound for hemodynamic assessment in children are limited, however, the two primary indications are considered the identification of pericardial tamponade and the assessment of hemodynamic status, on one hand studying left ventricular function and on the other hand assessing volume status by respiratory variation of the inferior vena cava. Then, some limitations should be encountered: specific expertise should be preferred, due to the wider range of structural and anatomic abnormalities in congenital heart disease than in adult heart disease, which may increase inter-operator variability in the case of low expertise with children; moreover, children may be less cooperative for the examination requirements compared to adults [38].

### 3.3. Venous Ultrasound: New Markers of Fluid Status

The quantification of significant venous congestion could also be performed by the VExUS grading system, considering that large veins (vena cava) as well as abnormal venous waveforms connected with the limit of the systemic venous compliance in the portal vein, hepatic veins and intrarenal veins have been associated with the congestive process, and with adverse consequences of venous hypertension. In states of intravascular volume overload the limits of venous compliance are reached; as a consequence, the normal dampening of the venous pulse due to the compliant nature of the smaller veins is lost, and the pulsations are transmitted back into the smaller veins.

The VExUS grading system is based on the evaluation of IVC diameter and venous Doppler waveform of the portal, hepatic and interlobular renal veins. In the presence of a IVC > 2 cm, each of these veins are evaluated and assigned to be representative of either being normal, mild congestion, or severe congestion. Hepatic Doppler is considered severely abnormal when the systolic (S) component is reversed (towards the heart) than the diastolic (D) component. Portal Doppler is considered severely abnormal when a variation in the velocities during cardiac cycle of ≥50% is seen. Intra-renal venous Doppler is considered severely abnormal when it is discontinuous with only a diastolic phase seen during the cardiac cycle.

**Figure 5:**
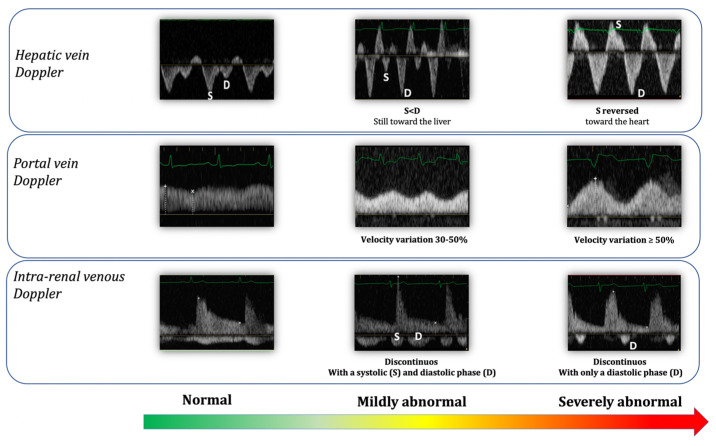
Normal findings and different grades of abnormal findings in venous ultrasound evaluation. Adapted from [39].

VExUS grade 0 is with no sign of congestion in any organ, VExUS grade 1 is with only mild congestive findings, VExUS grade 2 is with severe findings in only one organ, and VExUS grade 3 is with severe congestive findings in at least two of three organ systems (Table 2). Moreover, new grading systems are object of study [39] for a more complex phenotyping of patients according to VExUS mild or severe findings by hepatic, portal and intra-renal venous Doppler (appearing as shown in Figure 5).

The VExUS score should be of interest as a guide to the daily decision-making about fluid balance management in critical ill patients, even if further studies should aim to validate this grading system in different clinical settings. Moreover, the fact that each of the proposed markers has some limitations should be considered: hepatic vein Doppler is strongly connected with TR, which may influence its interpretation; pulsatile portal vein flow and IVC dilatation have been reported in healthy athletic volunteers; and intra-renal venous Doppler is more technically complicated to perform [8,39].

### 3.4. Lung Ultrasound

Lung ultrasound (LUS) can be extremely useful in patients in the CICU as it provides, in patients with acute respiratory failure and hypotension/shock, a point-of-care evaluation of pulmonary congestion, lung consolidation, pleural effusion, and pneumothorax [40].

Lung ultrasound can be performed with the investigation of three or four zones for B-lines on each hemithorax. Vertical B-lines provide a graded measure of interstitial or alveolar oedema with high interoperator reproducibility. At least three B-lines in two or more intercostal spaces bilaterally are considered indicative of interstitial or alveolar oedema in the acute care setting (Figure 3).

Despite their high accuracy in the identification of pulmonary oedema in patients with suspected AHF, B-lines can also be found in other conditions, such as interstitial lung disease, acute respiratory distress syndrome, pulmonary contusions and pneumonitis [4,29,41]. Nevertheless, generally in cardiogenic pulmonary edema the pleural line usually appears of normal morphology, with conserved pleural sliding, while B lines are dense, symmetrical and often confluent, with a gravimetric gradient. Moreover, B lines can be associated with bilateral pleural effusion; it stands out for its distribution and quality, so it appears as a homogeneous or inhomogeneous anechoic area. In particular, the echogenicity depends on the composition: the transudate, due to its prevalent fluid content, appears as a simple effusion mainly anechoic and homogeneous; while the exudate appears with fibrins and multiple echogenic spots [42,43]. Furthermore, in the suspect of pneumothorax, LUS must be performed with the patient supine, on the parasternal line and bilaterally: in this case, B lines, pleural sliding and lung pulse are absent; while we may find horizontal A lines, lung points and barcode sign [41,43,44].

LUS is also useful for follow up and the prognostic stratification of patients and can help the clinician to guide the management of the therapy; in fact, B lines have shown to reduce with diuretic therapy, and their number it is directly proportional to the BNP and the NYHA class [45]. Thus, LUS association with transthoracic echocardiography adds important information for the assessment of the degree of decompensation, both in terms of hemodynamic congestion and of pulmonary congestion [2]. Nevertheless, there are some limitations of LUS that are essentially patient-dependent: obese patients with their thickness of the ribcage; and subcutaneous emphysema or large thoracic dressings alter or preclude the propagation of the ultrasound and make the exam more challenging [40].

## 4. Pulmonary Hypertension

### 4.1. Assessment in Intensive Care Unit

Pulmonary hypertension (PH) is a common finding in patients admitted to the cardiac intensive care unit and is associated with worse prognosis compared to controls [46]. In critically ill patients with PH, such as those hospitalized for acute RV failure, survival is even poorer, with the rate of death or urgent transplantation estimated at 38% to 42% by 90 days [47,48].

Actually, PH is defined as an increase in mean pulmonary arterial pressure (mPAP) > 20 mmHg at rest, measured by right heart catheterization (RHC) [49]. Further evaluation of pulmonary vascular resistance (PVR) and pulmonary arterial wedge pressure (PAWP) is essential for the discrimination of pre- and post-capillary PH, in order to detect the cause of PH. The left heart disease is the most common cause of PH, whose frequency increases with severity of left-sided valvular disease. In these patients, a post-capillary PH, hemodynamically defined as mPAP > 20 mmHg and PAWP > 15 mmHg, is detected, either isolated or combined with a pre-capillary component (respectively, with PVR ≤ 2 Wood units (WU) or PVR > 2). Hemodynamic findings of mPAP > 20 mmHg, PAWP ≤ 15 mmHg and pulmonary PVR > 2 defines pre-capillary PH, suggesting a diagnosis of pulmonary arterial hypertension (PAH), PH from lung disease or from pulmonary artery obstruction (thrombo-embolic PH or other obstruction). Not rarely, multiple causes of PH co-exist and identifying the potentially multiple factors that are contributing is necessary for optimal management.

Although different groups of PH vary in terms of etiologies and pathogenesis, there is a common evolution of the disease, resulting in RV dysfunction and ultimately RV failure, which is an important predictor of survival in patients with PH. Thus, the optimal management of critically ill patients requires an understanding of RV function, the appropriate monitoring and identification of RV failure, and a physiologic approach to the optimization of volume status, RV afterload, and cardiac function.

**Figure 6:**
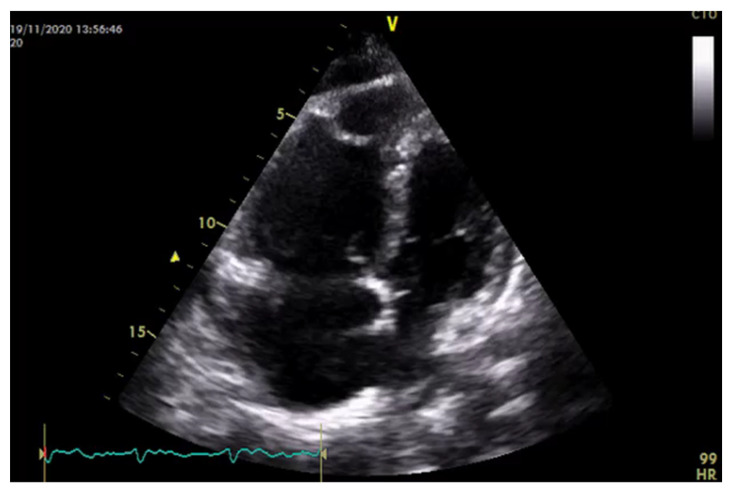
A 4-chamber view of standard echocardiography in a patient with PH. As results of the increased afterload, RV wall hypertrophies and dilates, developing a spherical shape accompanied by increased RV wall stress, RV dysfunction and severe RA dilatation.

RV failure, also referred as right HF, is defined as low CO and/or elevated right-sided filling pressures due to systolic and/or diastolic RV dysfunction. It often begins with a significant increase in RV afterload (pressure and/or volume overload), which leads to RV remodeling. RV consist of a thin wall and low volume/surface area, and shows greater compliance than the muscular thick-walled left ventricle. As results of the increased afterload in PH, RV wall hypertrophies and eventually dilates, developing a spherical shape accompanied by increased RV wall stress and RV dysfunction (Figure 6).

Systolic right-sided HF results in LV underfilling and low cardiac output, which impairs tissue perfusion and oxygenation. Moreover, LV filling and function is also impaired due to ventricular interdependence. Indeed, RV dilation causes the interventricular septum to shift towards the LV (“fattening”), which persists through the cardiac cycle, with consequently LV end diastolic volume reduction and LV ejection impairment [50]. The combination of diminished RV blood supply with increased oxygen demand due to hypertrophied RV contribute to worsen RV dysfunction.

The evaluation of RV function by transthoracic echocardiography remains the most valuable and useful tool, especially in critically ill patients, for diagnosis and monitoring patients with PH [51]. Despite alone it is insufficient to confirm a diagnosis of PH, which requires RHC, echocardiography can provide an estimation of hemodynamic parameters, evaluate right and left heart morphology, identify congenital heart disease or left heart disease as the underlying cause of PH [52]. In the recently published guidelines on the management of PH, peak tricuspid regurgitation velocity (TRV) has emerged as the key variable for assessing the echocardiographic probability of PH, more reliable than estimates of systolic pulmonary artery pressure (sPAP) [53]. Indeed, estimated sPAP at rest has shown to be not prognostic and irrelevant to therapeutic decision-making [54,55]. Conversely, in a population of 278 patients referred for PH, only tricuspid regurgitant velocity (TRV) > 2.8 m/s independently predicted PH, although this parameter is prone to underestimation, as in patients with severe TR [56], or overestimation, as in patients with high CO or in the case of the incorrect assignment of a peak TRV. In general, a peak TRV> 3.4 m/s suggests the high probability of PH, that needs to be interpreted in a clinical context and confirmed with further testing, including RHC. The TRV threshold of 2.8 m/s has been set to define the low (≤2.8 m/s) or intermediate (2.9–3.4 m/s) probability of PH [50]. The presence of additional variables related to ventricles morphology (e.g., RV/LV basal diameter/area ratio > 1.0 or flattening of the interventricular septum), pulmonary artery dilation and RV outflow tract (RVOT) blood flow or inferior vena cava and right atrial dilation can alter the level of echocardiographic probability of PH [50]. Some of these parameters have shown high accuracy in the diagnosis of PH and in predicting RV dysfunction, such as RVOT acceleration time < 105 ms with mid-systolic notching, which may suggest pre-capillary PH [57], or TAPSE/sPAP ratio < 0.55 mm/mmHg, which represent a non-invasive measure of RV-PA coupling [58]. Among echocardiographic parameters that deserve to be considered in the assessment of PH, TAPSE < 18 mm, tricuspid annulus velocity (S′ wave) derived from tissue Doppler imaging, <9.5 cm/s and RV FAC < 35% should be considered markers of RV dysfunction [50,59].

Recently, ventricular strain has been implemented to assess regional and global RV function [50,60] and to predict prognosis in PH patients. In a population of 44 patients affected by precapillary PH, Puwanant et al. demonstrated that chronic RV pressure overload directly affects RV longitudinal systolic deformation and interventricular septal and LV geometry [61]. RV strain reduction in patients with PAH was also described by Sachdev et al., that reported a greater degree of clinical deterioration within 6 months in those with RV free wall strain worse than −12.5% [62]. In a larger population of patients with PH of different etiologies, impaired RV longitudinal peak systolic strain (≥−19%) was significantly associated with worse New York Heart Association functional class, lower TAPSE excursion, and all-cause mortality at 2.6-years follow-up [63]. Real-time three-dimensional echocardiography (RT3DE) also represents a useful tool for the assessment of RV, able to accurately estimate RV size and EF and improve the assessment of RV function non-invasively [52]. The use of intravenous contrast agents can further improve RV visualization, particularly in smaller ventricles. In addition, RT3DE seems particularly useful to better understand specific causes of PH (i.e., septal defects, complex congenital pathology, left-sided valvular or ventricular heart disease) and to investigate RV functional and morphologic changes [64,65,66]. In this regard, a RT3DE study in 141 consecutive patients demonstrated that different causes of PH may lead to diverse RV remodeling, regardless of RV systolic pressures at rest [66].

Moreover, further attempts to find surrogates of RHC measures has been made in the last years, leading to the evaluation of two parameters i.e., echocardiographic Pulmonary to Left Atrial Ratio (ePLAR) and the ratio between tricuspid regurgitation and the velocity time integral assessed on RVOT (TRV/VTI RVOT).

ePLAR has been found to be a noninvasive parameter to distinguish pre-capillary and post-capillay PH, and is calculated as maximum tricuspid regurgitation continuous wave Doppler velocity (m/s) divided by the transmitral E-wave:septal mitral annular Doppler Tissue Imaging e’-wave ratio. Its normal values were estimated as 0.30 ± 0.09 m/s. Pre-capillary PH patients were found to have higher values, while post-capillary PH patients had lower values than the reference in a big study comparing the echocardiographic measures with invasive measures obtained by right heart catheterization [67].

On the other hand, TRV/VTI RVOT has been found to be a noninvasive parameter to estimate PVR augmentation. Particularly, it has showed to be a good screening tool to assess PH and PVR> 2 WU compared with invasively assessed PVR; however, it has showed less accuracy for the assessment of high PVR [68], above all in, particular clinical settings (such as chronic pulmonary embolisms [69]).

### 4.2. Possible Impact of Ventilation on the Right Ventricle

The echocardiographic assessment of RV function and the estimation of sPAP remains difficult and challenging, especially in patients under mechanical ventilation (MV). In this circumstance, insufflation increases intrathoracic chest pressure resulting in a decreased venous return, reduction in RV preload and increase in RV afterload. This translates into a reduction in RV stroke volume and the development of paradoxical septal bowing, which in turn results in decreased LV SV and CO [70]. Positive pressure ventilation may also impact on pulmonary circulation, by distending alveoli and compressing alveolar blood vessels, thus increasing PVR, which concurs to increase RV afterload. The concomitant decrease in systolic blood pressure may contribute to cardiac output decrease, by reducing coronary perfusion pressure to the RV [71]. In addition, all anesthetic agents used for the induction and maintenance of general anesthesia have varying degrees of impact on systemic vascular resistance and cardiac contractility and can, hence, potentially impair RV function [72]. In a prospective study on 53 patients undergoing anesthesia and positive pressure ventilation, a significant reduction of TAPSE and free wall strain was described, without changes in RVEF and SV measured with 3D echocardiography [73].

In patients undergoing MV echocardiographic an estimation of sPAP is also tricky. The increase in pulmonary systolic pressure observed during MV results in higher tricuspid and pulmonary regurgitation velocities during mechanical insufflation. Moreover, neither the size nor the variation of the IVC during MV are reliable variables in the estimation of right atrial pressure (RAP) [74]. In their study, Fisher et al. demonstrated a weak correlation between Doppler and the invasive evaluation of sPAP, suggesting a possible relation with an inaccurate RAP estimation [75]. This was confirmed by Vieillard-Baron et al. [76], who found a weak correlation between the noninvasive evaluation of central venous pressure (CVP) using IVC assessment and the invasive CVP. In this regard, some authors proposed to estimate sPAP by adding CVP, measured using a central venous catheter, to the peak TVR gradient measured by echocardiography, demonstrating a good correlation with invasive pressures [63]. Alternatively, the method of adding 10 mm Hg to the Doppler recorded RV-RA maximal pressure gradient, and has also been shown to be an accurate way to assess sPAP and to diagnose PH [77] and could be recommended in the absence of a central venous catheter to measure CVP.

It should be noted that studies that examined the outcomes of patients with PH in the ICU reported higher in-hospital mortality and longer hospital stays in those treated with MV [78]. Thus, according to current guidelines, intubation in patients admitted in the ICU with PH should be avoided whenever possible, due to the high risk of acute HF and circulatory collapse [50].

## Figures and Tables

**Table 1:** Echocardiographic indices of perfusion and congestion in patients in CICU, detailed on the basis of their utility and normal values or ranges.

		Utility	Normal Values or Ranges
Perfusion parameters	LVOT VTI and CI	A reliable quantitative parameter that truly reflects circulatory status and global end organ perfusion	LVOT VTI ≥ 18 cm CI ≥ 2.5 L/min/m^2^
	LV GLS	A valuable and sensitive tool for follow-up examinations	LV GLS < −20%
	RVOT VTI	Gives information about the right ventricle function and pulmonary vascular resistance	RVOT VTI ≥ 12 cm
	RV 2D FAC	Provides an estimate of global RV systolic function	RV FAC ≥ 35%
	TAPSE	Represents a measure of RV longitudinal function	TAPSE ≥ 17 mm
	TDI S’	Correlates well with other measures of global RV systolic function	S’ ≥ 9.5 cm/s
	Global longitudinal RV free wall strain	Useful for estimating RV global and regional systolic function	RV free wall strain < −20%
	LVOTO	Its diagnosis leads to drastic changes in the clinical management	LVOTO < 30 mmHg
Congestion parameters	IVC diameter	Used to estimate RA pressures	IVC < 21 mm that collapses > 50%
	TDI e’	Associated with increase mortality in the critically ill patients	Avg e’ ≥ 10 cm/s lateral e’ < 8 cm/s
	E/e’	A load-independent marker of LV filling pressure	E/e’ < 15
	PASP	The most used non-invasive tool for estimating PAP	PASP > 35 mmHg
	PAAT	Represents pulmonary flow acceleration, which increases as the vascular resistance is augmented	PAAT 136–153 ms
	PALS	An additional index of congestion to optimize therapeutic management	PALS ≥ 15%

CI, cardiac index; IVC, inferior vena cava; LV GLS, left ventricular global longitudinal strain; LVOTO, left ventricular outflow tract obstruction; LVOT VTI, left ventricular outflow tract velocity time integral; PAAT, pulmonary artery acceleration time; PALS, peak atrial longitudinal strain; PASP, pulmonary artery systolic pressure; RV, right ventricular; RV 2D FAC, RV 2 dimensional fractional area change; RVOT VTI, right ventricular outflow tract velocity time integral; TAPSE, tricuspid annular plane systolic excursion; TDI e’, tissue doppler imaging e’ wave; TDI S’, tissue doppler imaging S wave.

**Table 2:** Different grades of the venous excess ultrasound (VExUS) score obtained combining inferior vena cava (IVC) diameter and venous Doppler waveform of the portal, hepatic and interlobular renal veins.

VExUS Score	
Grade 0	IVC < 20 mm
Grade 1	IVC ≥ 21 mm
	Normal patterns or mild findings
Grade 2	IVC ≥ 21 mm
	Severe finding in only one organ
Grade 3	IVC ≥ 21 mm
	Severe findings in multiple organs

## Data Availability

Not applicable.
